# Does the health information system in Jordan support equity to improve health outcomes? Assessment and recommendations

**DOI:** 10.1186/s13690-024-01269-6

**Published:** 2024-04-12

**Authors:** Ahmad H. Alnawafleh, Hoda Rashad

**Affiliations:** 1 Faculty of Nursing, Mutah University, 61710 Mutah, Jordan; 2 Social Research Center, American University in Cairo, 11835 Cairo, Egypt

**Keywords:** Health inequality, Social determinants of health, Equity, Health information system, Jordan

## Abstract

**Background:**

This study is based on extensive evidence-based assessments. The aim of this paper is to evaluate how well Jordan’s health information system (HIS) incorporates social determinants of health inequity (SDHI) and to propose suggestions for future actions.

**Methods:**

An extensive evidence-based assessment was performed. A meta-synthesis of the inclusion of the SDHI in the HIS in Jordan was conducted. After searching and shortlisting, 23 papers were analyzed using Atlas.ti 9.0 employing thematic analysis technique.

**Results:**

The HIS in Jordan is quite comprehensive, comprising numerous data sources, various types of information, and data from multiple producers and managers. Nevertheless, the HIS confronts several obstacles and fails to ensure the timely and secure publication of available data. The assessment of the inclusion of the SDHI in the HIS showed that the HIS allows for the measurement of progress in relation to social policies and actions but has a very limited database for supporting the inclusion of health inequity measures. One reason for the difficulty in identifying fairness is that certain crucial information necessary for this task cannot be obtained through the available institutional HIS or population survey tools. Additionally, relevant modules for fairness may be missing from population surveys, possibly due to a failure to fully utilize the capabilities of the institutional HIS.

**Conclusion:**

There are opportunities to make use of Jordan’s dedication to fairness and its already established strong HIS. Some social determinants of health exist in the HIS, but much more data, information, and effort are needed to integrate the SDHI into the Jordanian HIS. A proposal from a regional initiative has put forward a comprehensive set of indicators for integrating SDHI into HIS, which could aid in achieving health equity in Jordan.

**Table:** 

Text box 1. Contributions to the literature
• Indicators for social determinants of health aid in achieving health equity.
• Including indicators for inequality and social determinants of health in the HIS is important.
• These indicators may enable clinicians to focus on less privileged populations to provide them with the required health services.

## Background

A framework that focuses on the social determinants of health acknowledges that factors beyond the healthcare system within society contribute to health disparities [1–4]. These disparities are influenced by variations in the environment, community, family, and individual levels. The framing of social determinants of health inequities (SDHI) differs from that of social determinants of health (SDH) in two key features [2, 5]. The SDHI places great importance on the examination of inequality and recognizes that various factors contribute to the determinants of health [2]. The SDHI proposes that disparities in health outcomes among social groups stem from inequitable distributions of social, economic, and cultural resources, as well as unequal access to opportunities for improving their circumstances. According to the principles of SDHI, the problem of unequal access to health resources can be traced back to larger political and economic systems and policies, as well as social factors and public services [2, 3]. This inequality is evident in the way society is divided into groups based on factors such as wealth and education and in how policies and practices favor certain groups over others in meeting their health needs.

Health information should first perceive and critically examine the occurrence of health disparities and any clustering among marginalized and underresourced communities. The distribution of health inequalities by social stratification should also be investigated, and this distribution should be linked to the fairness and responsiveness of macrostructures, policies, intermediate forces, and public services. An absence of acknowledgment of inequities and their consequences for health distribution could sustain them and might lead to their propagation. This could widen the health divide by disproportionately providing resources and opportunities to nonmarginalized groups that have already received a relatively greater share of health-related benefits. More importantly, acknowledging inequities supports a movement from targeting the disadvantaged to changing the distribution of their disadvantage. It supports the adoption of transformative, fair, and responsive policies and actions [2, 3]. The focus on SDHI to decrease health inequalities helps to realize development anchored on social justice and human rights and on ‘Leaving no One Behind’, as pursued by the Sustainable Development Goals (SDGs) [3, 6].

A HIS that follows the SDHI framework caters to a diverse group of people at various levels [2]. At the level of health professionals, the HIS should offer insight and facilitate effective communication, which can aid in diagnosing health issues and taking appropriate actions within local communities. If the HIS is successful in providing this support, health professionals can become more compassionate toward their patients and deliver health services that are responsive to the context of SDH [7]. This is especially important when navigating obstacles to accessing healthcare and other services during direct interactions with health services. Moreover, it is also valuable for implementing comprehensive social and health interventions at the community level. At the policy and public sector level, such a system can motivate the adoption of strategies that promote health equities in all policies (HEiAP) and intersectoral actions for health [2, 3, 7]. It can help promote well-being and health equity as a measure of development and social success.

The significance of incorporating the SDHI into Jordan’s HIS is acknowledged in this paper, which delves into the subject and provides suggestions for future actions. A thorough examination of pertinent resources and materials was conducted to produce this paper. Initially, the research methodology employed is outlined, followed by a depiction of Jordan’s HIS. An evaluation of the HIS and Jordan’s initiatives to enhance it ensues. The paper concludes with a discussion of the integration of SDH and health equity into the information system, along with an analysis of the results and recommendations for future steps.

## Methods

To gain a better understanding of how social factors contribute to health inequalities within Jordan’s HIS, a thorough examination of evidence was conducted. This involved conducting a comprehensive search of various databases and websites between June and August 2021, including scientific and gray literature sources such as www.google.jo. The search focused on specific keywords related to SDH, electronic medical records, HISs, health equity, health inequality, and Jordan. The authors have access to a wide range of databases and reports. Through this access, they reviewed a large volume of gray literature, including a wide range of relevant articles, mini-reviews, editorials, book chapters, newspaper articles, unpublished thesis papers, and nonscientific articles. The next step involved scanning and reading the article abstracts and content tables to determine which papers were relevant and should be included. All duplicates and studies not related to the Jordan HIS were excluded.

The method used to analyze the papers and reports that met the criteria involved employing the thematic analysis technique with the support of Atlas.ti 9.0 software. This approach involves identifying and organizing themes from the data, analyzing them, and presenting a summary. One benefit of using thematic analysis is that it enables researchers to gain a deeper understanding of the themes that emerge. This process is particularly useful for summarizing a large amount of data and literature in a structured and organized way.

## Results

With support from contacts in relevant departments at the Ministry of Health (MoH) in Jordan and other agencies involved in the HIS, the authors managed to collect over 120 documents. Various research techniques, such as literature reviews, theoretical research, technical reports, case studies, and other qualitative, quantitative, and mixed methods or approaches, were utilized to identify relevant studies or reports. After eliminating duplicates and articles that did not meet the literature foci, 76 articles were selected. During the full reading of the articles, 53 papers were excluded because they were deemed unsuitable for the meta-synthesis. After this sorting process, 23 papers were selected for analysis and uploaded to Atlas.ti 9.0, which is software used for data analysis and synthesis.

The HIS in Jordan is well organized and encompasses a wide range of data sources, information types, and individuals responsible for producing and managing the data. Certain data tools used involve physical forms that are subsequently verified, coded, entered, and processed. Additionally, electronic records and completely automated systems are utilized for certain data types and by some data management systems.

Currently, in Jordan’s HIS, the evaluation of various types of data shows that birth registrations are almost complete (99%), while death registrations represent 75%. According to Khader, Alyahya et al. [8], some neonatal deaths and stillbirths are not fully registered due to a malfunctioning reporting system that places the responsibility of registration on families [9].

The quality of reporting for the cause of death statistics in Jordan does not meet the quality-of-care standards. According to the SCORE data report, only 59% of total deaths in Jordan were accompanied by a medical certificate with the cause of death (MCCD) and ICD coding [10]. The accuracy of the certificates in determining the cause of death varied between 20% and 29%, according to Alyahya et al.‘s [11] research. This study emphasizes the need for ongoing training of Jordanian healthcare practitioners in utilizing the ICD-10 coding system when implementing new health technology, as well as the relative novelty of electronic health records (EHRs) and ICD-10 use in public hospitals in Jordan [12, 13]. The research suggests that the current program is not standardized and lacks the necessary coding system to precisely identify and record the causes of death [11].

Electronic medical records in the field of obstetrics lack a comprehensive template for a record because it sometimes does not track the patient’s entire medical journey [14–17]. This is because records were established to record patient visits from the initial care provided during admission to the emergency department through the triage process, and the process of assessing and managing patients in labor wards to any destination should be documented until the treatment is completed [15, 16].

## The inclusion of SDHI in the information system

To provide information on health inequalities and the underlying social factors, an information system with an SDHI framework should be implemented. This system should also provide data on the fairness of upstream policies and public services that shape the social factors of health inequalities [2]. In Jordan, the institutional HIS (see Table 1) is rich in data on health inequalities and their social underpinnings, but it allows for measurements based only on geographic stratifiers and not other social stratifiers, such as gender and age. Population-based (see Table 2) surveys and submodules designed for this purpose are valuable for producing knowledge and recommendations on health inequalities and their social determinants [18, 19].

**Table 1:** Institutional health information system

Data sources and references	Owners of the data	Data production	Program	data collected	Forms of data
Institutional data [8, 10, 19, 22, 24, 25, 29–32]	MoH, RMS, Private sector, University	Routinely	Individual Service and Source Records	Institutional data	Paper based
National electronic registration system [17, 19, 23, 24, 31, 33, 34]	MoH, RMS, Private sector, University	Routinely	Hakeem	Information gathered from individual cases at the healthcare facility level is compiled and organized at the levels of the healthcare facility, governorate, and nation.	Computerized
National electronic registration system [19]	MoH	Routinely	Interactive Electronic Reporting System (IERS)	Information gathered from individual cases at the healthcare facility level is compiled and organized at the levels of the healthcare facility, governorate, and nation.	Computerized (tablet based)
Institutional data [17, 19, 23, 24, 31, 33, 34]	Individual institution	Routinely	Individual, service and resource records	Institutional data	Paper and computer based
National registration system [12, 13, 19, 24, 27, 29, 31, 32]	Directorate of NCDs (MoH)	Routinely collected and collated annually	National Renal Disease Registry	Patients are registered upon receiving a confirmed diagnosis at healthcare facilities and specialized hospitals, with their information classified according to three factors: age, sex, and place of residence.	Paper based at Hospital level – Collated and digitized centrally
National registration system [12, 13, 19, 24, 27, 29, 31, 32]	Directorate of NCDs (MoH)	Routinely collected and collated annually	National Thalassemia Registry	Patients are registered upon receiving a confirmed diagnosis at healthcare facilities and specialized hospitals, with their information classified according to three factors: age, sex, and place of residence.	Paper based at Hospital level – Collated and digitized centrally
National registration system [12, 13, 24, 27, 29, 31, 32]	Directorate of NCDs (MoH)	Routinely collected and collated annually	National Cancer Registry	Patients are registered upon receiving a confirmed diagnosis at healthcare facilities and specialized hospitals, with their information classified according to three factors: age, sex, and place of residence.	Paper based at Hospital level – Collated and digitized centrally

*Source* Study data sources

**Table 2:** Population health information system

Data sources and references	Owners of the data	Data production	Program	data collected	Forms of data
Census [8–10, 18, 22, 24, 25, 27, 29, 32]	Directorate of Public Statistics	Every 10 years	1. Social, economic and demographic conditions locally and nationally. 2. Children and people living with disabilities	Population based data	Paper based, digitized at central level (DoS)
Surveys [18]	Directorate of Public Statistics	Every 5 years	the Demographic and Health Survey (DHS)	Data layers include several categories, such as age groups, marital status, area of residence (whether urban or rural), governorate level, nationality, education level, and wealth quintiles.	The paper documents are converted into digital form at a centralized location, which is managed by the Population Higher Council.
Civil Registry of Vital Statistics (CRVS) [10, 19, 22, 25, 32]	Ministry of interior	Routinely	Data regarding the registration of births and deaths obtained from the civil registry of vital statistics (CRVS)	1. Data on Births 2. Marriage 3. Divorce/ Separation 4. Deaths	Centralized digitization and paper-based formats are both utilized.
National Health Accounts [18, 19, 30–32]	Higher Health Council	Annually	Accounts of National Health	Health indicators accounts	Paper based and Email collected and collated centrally

*Source* Study data sources

Surveys are valuable because of their ability to link individual-level data with different social determinants across multilevel pathways of influence. This includes how social stratifications in society are reflected in the proximate level of behaviors, livelihoods, and community contextual forces.

An example of the value of population surveys in investigating health inequalities and their social determinants is available in two recent studies [14, 20]. These studies used data provided by the Jordan Population and Family Health Survey (JPFHS) of 2012 and 2017, respectively. These studies have investigated inequality challenges in Jordan and have produced important analyses, findings, and recommendations related to health inequalities and their social underpinnings.

The analysis of the 2012 JPFHS indicated that the summary inequality measures by geographic and gendered cultural context stratifiers are relatively small compared to those of the wealth stratifiers and four other Arab countries (Egypt, Morocco, Oman, and Sudan) [14]. The rich data from the 2017 JPFHS have allowed a more comprehensive investigation of inequalities and their trends [18]. The investigation covered additional dimensions of health and added nationality as a social stratifier that is quite relevant to the Jordanian context. The rich data provided important findings covering child health and wellbeing, adult health and noncommunicable diseases (NCDs), sexual and reproductive health, and health sector performance and capacity [20]. Other earlier studies indicated that Jordan, in comparison to other countries with similar per capita gross domestic product (GDP), exhibits low inequality [21, 22]. Such inequalities have been at a low level since 2006, with fluctuations during recent decades [21]. The more recent past, however, is pointing to an increase in health inequalities [14].

In terms of the data to investigate the fair responsiveness of public services, special modules can be incorporated in a population-based survey to collect data that allows such an investigation [10, 18]. Additionally, the potential of institutional HIS could be more effectively capitalized on. These assessments (population and institutional surveys) revealed the limitations of the HIS and that very few studies have performed such investigations. One of the studies used the JPFHS of 2017 to integrate a module on health system capacity and performance in the data collection instrument [20, 23]. This allowed important findings that showed the severe inequitable distribution of health sector capacity, insurance coverage, and health sector performance by four stratifiers [20]. The inequalities in health service provision by the level of education, employment, income, residence area and other dimensions were also indicated in a World Health Organization and Regional Office for the Eastern Mediterranean study. The World Health Organization and Regional Office for the Eastern Mediterranean [22] emphasized that institutionally based HIS data at the subnational level in Jordan would allow measurement of area inequality for the provision of health services.

The only consistent sources of information on the quality and extent of health services in Jordan are the HIS and Civil Registry of Vital Statistics (CRVS) systems, which are based on institutions [20, 24]. Nevertheless, there is no conclusive proof that this information can accurately measure health equity with regard to social stratifiers other than geographic classifications.

The ability of the HIS in Jordan to measure progress in SDH policies and actions was inferred from the application of a global assessment tool (SCORE) performed by the MoH and relevant stakeholders in Jordan [10]. The WHO and its collaborators created a SCORE for the health data technical package with the aim of supporting Jordan’s data systems and abilities in tracking advancements toward the SDGs pertaining to health, such as Universal Health Coverage (UHC), health priorities and objectives at the national and subnational levels [6, 19]. According to the World Health Organization [25], the SCORE assessment showed that progress on SDH has been achieved. The World Health Organization [10] reported that 81% of health indicators have data available to monitor health-related SDGs. The capacity of the system is mostly moderate in the process of monitoring the SDGs based on the availability of the latest data [6].

The inability of the HIS to provide the necessary data to investigate inequities in policies is explained by the fact that the data needed to conduct the policy analysis for the investigation of inequities are not incorporated into the HIS in Jordan. This is particularly true since the information required at the policy level does not lend itself to the institutional HIS and population survey tools.

## Discussion

In Jordan, HIS evaluation reveals a complicated system that involves multiple data sources, various types of information, and numerous data producers and managers [26]. Unfortunately, the system does not guarantee the prompt availability of data or the timely release of information. Despite these issues, the HIS’s richness can be better utilized to promote health in Jordan and support its development plans [27]. The evaluation acknowledged the challenges in strengthening the HIS, but it also identified various recommendations and efforts to address them.

There is a need for legislation that mandates hospitals and other healthcare providers to promptly report births directly to the Department of Civil Status and Passports [13]. In essence, modifying the registration process by transferring responsibility from families to healthcare providers is necessary to achieve full registration of births and deaths [12]. To facilitate the registration process, an electronic system linking hospitals to the Department of Civil Status and Passports has been recommended [12, 22]. The HIS should gather all pertinent information about direct, indirect causes and contributing factors of death to accurately calculate statistics for cause of death. This will allow healthcare professionals and stakeholders to review and audit deaths to prevent future similar occurrences. The adoption of the International Classification of Diseases (ICD-10) coding system, based on the assessment of evidence, has various benefits [17, 22]. It enables healthcare providers to classify diseases, enhance the documentation of diagnoses and related complications, and effectively assess health care outcomes, especially in underprivileged and rural areas.

The inclusion of health inequity in the HIS was shown to be quite deficient, and it is not receiving the attention of the many efforts to strengthen the HIS. To effectively address health inequity in the HIS, it is necessary to collect data that go beyond measuring social disparities and identifying the policies responsible for creating unjust social stratification in society. These data should consider the equitable distribution of public services that respond to the diverse needs of various social groups.

The initial category of information can be collected from surveys conducted among the general population. In fact, the evaluation showed that the data obtained from the JPFHS provided comprehensive insights into the extent and trajectory of health disparities across various social groups [18].

Population-based surveys, capture new health challenges and new social drivers, yet the total reliance on them to replace institutional HIS is not efficient. This is explained by the periodic nature of these surveys and the fact that they do not make use of the richness and wide range of information provided in routine and more regular institutional sources of the HIS [1, 18]. There is a clear need to cross-link individual records with socioeconomic, behavioral and contextual determinants [24]. This would be possible through linking the CRVS, for example, with the geographic location and the population surveys. These cross linkages can support the investigation and monitoring of health inequalities and their social underpinnings.

The second type of data relating to the fairness of public policies can be based on the institutional information systems of different sectors, including the institutional HIS. These could be easily modified to measure the fairness of the provision of services to different social groups [10, 21]. However, linking the distribution of services to inequalities in health outcomes is more difficult, as it requires linking data on services, social stratifiers, and health outcomes.

The contributions of surveys to investigating inequities in public services rely on the introduction of additional well-concepted modules. These modules can be guided by the specialized health facility submodules added to the most recent Jordan Population and Family Health (JPFH) survey in Jordan [10, 18, 19, 21, 28].

The third type of data on the fairness of upstream policies and their impact is glaringly missing in the Jordan HIS. Similar to the majority of other countries, the absence of relevant data and information is reinforced by the fact that the mainstream of HEiAP has not been embraced by Jordan. The WHO has called for health in all policies (HiAP) to be replaced by HEiAP and to be implemented as a policy approach [2, 3, 14]. This automatically produces the type of data that the HIS currently lacks.

It should be emphasized, however, that the inclusion of the equity lens in the HIS requires additional conceptual and methodological innovations. These pertain to the identification of policies responsible for social stratification in society, the specifications of the data and information needed to investigate the fairness of these policies, the inclusion of these data in the proposed data repository, and the analytical and methodological skills to utilize these varied pieces of information to investigate the multilevel drivers of inequalities and the tracing of such inequalities to their root causes.

An important next step is to develop a database needed for applying the multilevel conceptual framework that integrates both the relevant social stratifiers with their manifestations in risks and opportunities for health, as well as their underpinnings of policies and public services. Currently, in the first phase of an ambitious activity, such a framework has been applied, and a detailed set of indicators that are needed to ensure the inclusion of SDHI in the HIS [2]. The next step is to adapt the proposed indicators to the context of Jordan to demonstrate its relevance to equity policies in Jordan and to illustrate their value in mainstreaming an equity lens in the implementation of programs.

## Challenges and limitations

The health system in Jordan is facing several challenges due to the lack of a national reference entity for research and health studies and the inadequate computerization of the health system [19, 21, 22, 26]. These challenges include weaknesses in modern electronic health system (E-Health) applications, limited access to private sector data and information, and difficulties formulating evidence-based policies and decisions [26]. To overcome these challenges, there is a need for a unified source of health information in Jordan, as the current data generated through different sources are not well integrated with the HIS, leading to gaps in its availability and readiness [19]. Furthermore, the HIS in Jordan lacks linkages and integration with routine information from various departments within the MoH and other ministries such as the CRVS [26].

## Recommendations

Three types of data are required for SDHI inclusion:

1. This study provides data and information on health inequalities that could be linked to their social underpinnings.
2. The data and information on the fairness of public policies could be linked to their consequences for health inequalities.
3. The data and information on the fairness of upstream policies could be linked to their impact on social stratification and on the equity of public services and social arrangements.

## Conclusions

Jordan recognizes the significance of fairness to people’s health and well-being and has a strategic commitment to equity. The country’s availability of a solid HIS foundation and the many efforts to strengthen it present an opportunity to help the country secure the needed evidence base to support development and well-being. Some data on social determinants of health are included in the Jordanian HIS, but much more data, information, and effort are needed to integrate the SDHI into the HIS of Jordan. The recently proposed package of indicators can be utilized to incorporate SDHI into HIS, and serve as the foundation for Jordan’s health equity policies and interventions. Incorporating this package into a comprehensive HIS and tailoring it to the country’s particular circumstances is essential.

## Data Availability

All data generated or analyzed during this study are included in this manuscript, in addition to the published articles and the supplementary information links below: Equity and Social Determinants of Health in Health Information Systems: https://www.aucegypt.edu/research/src/equity-health-information-system. Package of Indicators and Measures to Monitor Health Inequities and Guide Policies: https://documents.aucegypt.edu/Docs/src/Package%20of%20Indicators%20and%20Measures.pdf. Health Information System Strategic Plan 2019–2023: https://www.moh.gov.jo/ebv4.0/root_storage/en/eb_list_page/health_information_system_strategic_plan(2019-2023).pdf.

## Abbreviations

CRVS: Civil Registry of Vital Statistics
DHS: Demographic and Health Survey
DOS: Department of Statistics
HER: Electronic health records
ETCEA: Emerging Trends in Computing and Engineering Applications
GDP: Gross Domestic Product
HEiAP: Health Equity in all policies
HiAP: Health in all policies
HIS: Health information system
HMN: Health Metrics Network
ICD: International Classification of Diseases
IDRC: International Development Research Centre
JPFH: Jordan Population and Family Health
JPFHS: Jordan Population and Family Health Survey
JSANDS: Jordan stillbirths and neonatal deaths surveillance
MCCD: Medical certificate with the cause of death
MENAHIA: Middle East and North African Health Informatics Association
MoH: Ministry of Health
NCD: noncommunicable diseases
SDG: Sustainable Development Goals
SDH: Social determinants of health
SDHI: Social determinants of health inequity
UHC: Universal Health Coverage
